# Health of Louisville

**Published:** 1852-09

**Authors:** 


### HEALTH OF LOUISVILLE.

The health of the city has improved since we penned our remarks on the subject in our last number, and may now be pronounced excellent. Cholera, which has visited a number of the smaller towns in the State, seems to have disappeared wholly from Louisville, and dysentery which was prevalant in June and July, has so far abated, that we now rarely hear of a case. The tendency to fever seems also to be less than it was a month ago. Copious and timely showers visited the city and surrounding country during the month of August, and have been followed by a mitigation of the intense heat.

